# Lateral Ventricle Enlargement and Cortical Thinning in Idiopathic Normal-pressure Hydrocephalus Patients

**DOI:** 10.1038/s41598-018-31399-1

**Published:** 2018-09-06

**Authors:** Kyunghun Kang, Kichang Kwak, Uicheul Yoon, Jong-Min Lee

**Affiliations:** 1 Department of Biomedical Engineering, Hanyang University, Seoul, South Korea; 2 Department of Neurology, School of Medicine, Kyungpook National University, Daegu, South Korea; 3 McGill Centre for Integrative Neuroscience, Montreal Neurological Institute, McGill University, Montreal, Quebec, Canada; 4 Department of Biomedical Engineering, Daegu Catholic University, Gyeongsan-si, South Korea

## Abstract

We utilized three-dimensional, surface-based, morphometric analysis to investigate ventricle shape between 2 groups: (1) idiopathic normal-pressure hydrocephalus (INPH) patients who had a positive response to the cerebrospinal fluid tap test (CSFTT) and (2) healthy controls. The aims were (1) to evaluate the location of INPH-related structural abnormalities of the lateral ventricles and (2) to investigate relationships between lateral ventricular enlargement and cortical thinning in INPH patients. Thirty-three INPH patients and 23 healthy controls were included in this study. We used sparse canonical correlation analysis to show correlated regions of ventricular surface expansion and cortical thinning. Significant surface expansion in the INPH group was observed mainly in clusters bilaterally located in the superior portion of the lateral ventricles, adjacent to the high convexity of the frontal and parietal regions. INPH patients showed a significant bilateral expansion of both the temporal horns of the lateral ventricles and the medial aspects of the frontal horns of the lateral ventricles to surrounding brain regions, including the medial frontal lobe. Ventricular surface expansion was associated with cortical thinning in the bilateral orbitofrontal cortex, bilateral rostral anterior cingulate cortex, left parahippocampal cortex, left temporal pole, right insula, right inferior temporal cortex, and right fusiform gyrus. These results suggest that patients with INPH have unique patterns of ventricular surface expansion. Our findings encourage future studies to elucidate the underlying mechanism of lateral ventricular morphometric abnormalities in INPH patients.

## Introduction

Idiopathic normal-pressure hydrocephalus (INPH) is an uncommon neurological disorder of uncertain origin characterized by normal cerebrospinal fluid (CSF) pressure at lumbar puncture and a triad of symptoms including gait disturbance, cognitive impairment, and urinary dysfunction^1^. And enlargement of the cerebral ventricles is known to play a central role in the diagnosis of INPH^2^. There are markers of ventricular enlargement. First, the Evans’ index measures the maximal width of the frontal horns in relation to the inner diameter of the cranium^3^. Both the international and the Japanese guidelines define ventricular enlargement as an Evans’ index greater than 0.3^1,4^. It has been generally used as an indirect, surrogate marker of ventricular volume^5–7^. Second, volumetric measurements have been recommended to represent an accurate estimate of true ventricular size^8^. Volumetric assessment of lateral ventricles has been used to distinguish INPH patients from healthy subjects^2,9,10^. However, the volumetric approach also has limitations in providing specific anatomical information on regional changes^11,12^. Third, more recently, three-dimensional shape analysis of the lateral ventricles has been developed for a more refined detection of subtle changes in shape composition, which can be neglected in volumetric measurement^11,13^. And structural changes in the lateral ventricles have not been studied in INPH subjects in terms of shape deformation.

Measuring the thickness of the human cerebral cortex is of great interest in studies of neurodegenerative diseases^14^. And the thickness of the cortex can be a useful measure for understanding the disease^14^. For example, one study showed that an abnormally thin cortex may be associated with changes in gray matter that correlate with specific neuropathologies and neurological conditions such as Alzheimer’s disease (AD)^14^. Interestingly, widespread cortical thinning was also previously reported in animal models and children with hydrocephalus^15–17^. The INPH group in a study, when compared to controls, also exhibited cortical thinning in the middle temporal lobe^18^. Furthermore, hydrocephalus is characterized by enlargement of the cerebral ventricles and in animal studies, an increasing degree of ventriculomegaly is associated with more marked thinning of the cerebral cortex^15,17^. To our knowledge, however, an investigation of the relationship between lateral ventricular enlargement and cortical thinning in INPH patients has not been reported. Sparse canonical correlation analysis (SCCA), is a powerful bi-multivariate analysis technique^19,20^. SCCA provides a new window to interpret neuroimaging data, especially the relationship between cortical thickness measures and other neuroimaging measurements^21^.

The CSF tap test (CSFTT) is considered a valuable tool for the diagnosis of INPH and the prediction of shunt effectiveness in INPH patients^4^. Following the Japanese guideline, clinical improvement after the CSFTT is an important indicator that enhances diagnostic certainty from possible to probable^4^. In addition, shunt surgery is indicated for patients with INPH who exhibit a positive CSFTT response, and the CSFTT has a high positive predictive value for successful shunt surgery^4^. Therefore, there is consensus that the CSFTT is a key step in the diagnosis of INPH^4^.

In this study, we investigated the shape of the lateral ventricles utilizing a three-dimensional, surface-based, morphometric approach in 2 groups: (1) INPH patients who had a positive response to the CSFTT and (2) healthy controls. The aims of the study are (1) to evaluate the location of INPH-related structural abnormalities of the lateral ventricles and (2) to investigate relationships between lateral ventricular enlargement and cortical thinning in INPH patients. We hypothesized that INPH patients might show a characteristic pattern of lateral ventricular enlargement and that there may be unique relationships between lateral ventricular enlargement and cortical thinning in INPH patients.

## Methods

### Participants

Study participants were prospectively recruited from patients who visited the Center for Neurodegenerative Diseases of Kyungpook National University Medical Center, South Korea between June 2013 to September 2015. All participants gave written, informed consent for clinical evaluation and MRI. All INPH patients also consented for CSFTT. This study protocol was approved by the Institutional Review Board of Kyungpook National University Medical Center. All methods were performed in accordance with relevant guidelines and regulations. Diagnosis of INPH was made using the criteria proposed by Relkin *et al*.^1^. Patients had to be older than 40 years of age with an insidious progression of symptoms (gait disturbance plus at least one other area of impairment in either cognition, urinary symptoms, or both) for at least 6 months and have normal CSF opening pressure. Brain MRI of all INPH patients showed widening of the ventricles (Evans’ ratio > 0.3) and no macroscopic obstruction of CSF flow. Patients with stroke, other neurological, metabolic, or neoplastic disorders which might produce dementia symptoms or parkinsonism, a recent history of heavy alcohol use, or a history of hospitalization for major psychiatric disorder were excluded. No participant showed evidence of a related antecedent event, such as head trauma, intracerebral hemorrhage, meningitis, or another known cause of secondary hydrocephalus.

Criteria for the categorization of healthy controls were as follows: no active neurological, systemic, or psychiatric disorders; normal neurological examination; and independently functioning community dweller. Global cognition of the healthy control was also assessed by the Korean-Mini Mental State Examination (K-MMSE).

### Assessing illness severity

All INPH patients underwent comprehensive clinical scales as follows. Most of these scales were not assessed for healthy controls, because selection as a healthy control subject required a normal neurological examination. The patients’ severity of dementia and general cognitive state were evaluated by means of the K-MMSE and Clinical Dementia Rating Scale (CDR)^22,23^. The Frontal Assessment Battery (FAB), a tool designed for assessing frontal lobe symptoms, was used^24^. And the total FAB score ranges from 0 to 18. A higher score means a better performance. The Trail Making Test Part A (TMT-A), a common neuropsychological test to evaluate psychomotor speed, was also used^25^. The INPHGS, a clinician-rated scale to assess the severity of each fundamental symptom of INPH (cognitive impairment, gait disturbance and urinary disturbance) after an unstructured interview with patients and caregivers, was used^26^. The score of each domain ranges from 0 to 4. Grade 0 indicates normal and grade 1 indicates subjective symptoms but no objective disturbance. Grades 2, 3 and 4 indicate mild, moderate and severe disturbances, respectively. Gait assessment included measurements of time on the Timed Up and Go (TUG) test and 10 meter walking test^26–29^. In the TUG test, the time it takes a subject sitting in an armchair to stand up, walk forward 3 meters, and return to the seated position is measured. Features of gait disturbance related to INPH were estimated using the Gait Status Scale (GSS)^26^. This scale focuses on 8 factors of gait disturbance: (1) postural stability; (2) independence of walking; (3) wide base gait; (4) lateral sway; (5) petit-pas gait; (6) festinating gait; (7) freezing of gait; and (8) disturbed tandem walking. We used the total score of the 8 items of the GSS, which ranged from 0 to 16. A higher score reflects worse symptoms.

### Cerebrospinal fluid tap test

All INPH patients received a lumbar tap removing 30–50 ml of CSF. After the tap, the patients were re-evaluated using the INPHGS, which is a validated scale for measuring INPH symptom severity, and the TUG test. Gait changes were evaluated 1 day after the tap, while cognition and urination changes were evaluated at one week^30^. CSFTT responses were defined using these scales. The following criteria were used to identify responders: improvement of one point or more on the INPHGS or more than 10% improvement in time on the TUG test^4,30^.

### MRI imaging acquisition

MRI data were obtained using a 3.0 Tesla system (GE Discovery MR750, GE Healthcare). Three-dimensional T1-weighted, sagittal, and inversion-recovery fast spoiled gradient echo (IR-FSPGR) MRI images of the whole head, designed to optimally discriminate between brain tissues (sagittal slice thickness 1.0 mm, no gap, TR = 8.2 ms, TE = 3.2 ms, flip angle 12°, matrix size 256 × 256 pixels, and field of view = 240 mm) were acquired.

### Ventricle shape analysis

First, a hierarchical, multi-scale, non-linear fitting algorithm (ANIMAL) was applied to obtain the 3D deformation vector field that maps the template onto the individual brain volume. And then the resulting vector field was used to transform the ventricle atlas. These segmentations were processed to fill any interior holes and were separated into the left and right ventricle. When necessary, the segmentation was manually corrected using ITK-SNAP (www.itk-snap.org). Next, through the 3D spherical harmonic-based point distribution model (SPHARM-PDM)^31^, the processed binary segmentations of the left and right ventricle were converted to surface meshes, and a spherical parameterization was computed for the surface meshes using an area-preserving, distortion-minimizing spherical mapping. These SPHARM-PDM surface meshes are all spatially aligned using a rigid Procrustes alignment. After shape correspondence establishment and alignment, the ventricle difference was defined as the Euclidean distance between each subject and healthy control average shape.

### Cortical thickness

The following pipeline image processing steps were applied for further analysis, as described in detail elsewhere^32–34^. At first, the native MRI data of all subjects were registered into the template using a linear transformation and were corrected for intensity non-uniformity artifacts^35^. And then, an artificial neural network classifier was applied to gray matter (GM), white matter (WM) and CSF^34^. Finally, partial volume levels, MRI intensity mixing at the tissue interfaces due to the finite resolution of the imaging device, were estimated and corrected using a trimmed minimum covariance determinant method^36^. The surfaces of the inner and outer cortices were extracted automatically using the Constrained Laplacian-based Automated Segmentation with Proximities algorithm^33^. Cortical thickness was defined as the Euclidean distance between the linked vertices of the inner and outer surfaces; there were 40,962 vertices in each hemisphere in native space. The thickness value was spatially normalized using surface-based two-dimensional registration with a sphere-to-sphere warping algorithm. Thus, the vertices of each subject were nonlinearly registered to a standard space to compare thickness across subjects. Cortical thickness was subsequently smoothed using a surface-based diffusion kernel in order to increase the signal-to-noise ratio. We chose a 30-mm full-width at half-maximum kernel size to maximize statistical power while minimizing false positives^37^.

### W-score

We applied W-score mapping to identify the degree of cortical atrophy and ventricle expansion in each patient with a healthy control as a reference. Details on the theory and computation of W-scores are available elsewhere^38,39^. In this study, W-score maps were computed vertex-wise for the surface model of each imaging data set according to the following formula: W-score = [(INPH patient’s raw value) − (expected value in the healthy control)]/(SD of the residuals in the healthy control). W-scores are similar to Z-scores in that they have a mean value of 0 and a SD of 1 in the healthy control, and values of +1.65 and −1.65 correspond to the 95^th^ and 5^th^ percentiles, respectively.

### Sparse canonical correlation analysis

Because traditional canonical correlation analysis is severely limited when the dimensionality of the data is larger than the number of subjects, several studies used the SCCA approach for multivariate associations^21,40–44^. Avants *et al*. used SCCA to identify related patterns of white matter integrity and cortical thickness in AD and frontotemporal dementia^44^. And Jang *et al*. used SCCA to show correlated cortical thinning and WM microstructural changes in subtype dementia^21^. These studies used a SCCA to describe relationships between two sets of multi variables obtained from images. In other words, it simultaneously finds the canonical weight vectors in each data that maximize the correlation of the projections of each ventricle measurement and cortical thickness input data onto their canonical weight vectors. SCCA for each patient group was carried out using R packages (http://cran.r-project.org/web/packages/PMA/) with positivity and sparseness constraints^43^. The sparseness parameters, which control the sparsity for either set of the canonical variates, were selected as approximately half of the vertex dimension of the input data to focus on spatially distributed patterns. For visualization, canonical weight vectors of each data set were mapped on a standard surface model for cortical thickness and healthy control average template for ventricle measure. We used permutation testing of 2,000 iterations to assess significance of the SCCA. For the permutation test, we randomly reordered the possible pairs of the two input images (ventricle measure and cortical thickness). However, the two images were not selected from the same subject. The p-value for canonical variates was estimated as the ratio of ‘the number of the permutations in which correlation value exceeded the original correlation value’ to ‘the number of total permutations’. We considered a p-value of 0.05 as statistically significant in this study.

### Statistical analyses

The IBM SPSS Statistics for Windows version 21.0.0 was used for analyses of data. The demographic data were compared between the INPH and control groups. Fisher’s exact and chi-square tests were used to compare categorical variables, while the Student *t* tests and Mann-Whitney U tests were used to compare continuous variables. For the comparison of volume measurements of the lateral ventricles, analysis of covariance (ANCOVA) was used to examine the differences in ventricular volumes between INPH patients and healthy controls adjusting for age and intracranial volume (ICV). The ICV was analyzed by ANCOVA with age as a covariate. To compare mean cortical thicknesses across the entire vertices between groups, ANCOVA was performed, controlling for age and ICV. Statistical significance was set at p < 0.05.

The localized differences in surface expansion of lateral ventricles between the groups were analyzed using ANCOVA on a vertex-by-vertex basis with covariates of age and ICV. Statistical significance was assessed by Bonferroni correction at a p-value of 0.01 to correct the result for multiple comparisons. In these analyses, we applied this rather strict significance threshold to minimize false-positive effects.

## Results

Table 1 lists the demographic and clinical features for INPH and control subjects. There were no significant differences in the distributions of age and gender between the 2 groups. And, patients with INPH had significantly lower K-MMSE scores than the control subjects.

**Table 1:** Demographic data and clinical characteristics of INPH patients and controls at baseline. For INPH patients, data were collected before the CSFTT.

Characteristics	INPH (n = 33)	Control (n = 23)	*P* Value
Gender, male	21 (63.6)	9 (39.1)	0.070
Age (year)	73.5 ± 5.0	70.9 ± 4.3	0.059
Education (year)	9.2 ± 4.4	12.9 ± 4.5	0.005
Duration of symptoms (year)	2.5 ± 2.4		
K-MMSE	19.9 ± 6.9	27.1 ± 2.4	<0.001
CDR (0:0.5:1:2:3)	0:17:9:4:3		
INPHGS			
GS-Gait	1.6 ± 0.6		
GS-Cogn	2.7 ± 0.7		
GS-Urin	1.6 ± 1.2		
Total	6.0 ± 1.6		
TUG	21.3 ± 13.9		
10-meter walking test	20.1 ± 18.8		
GSS	7.5 ± 2.5		
FAB	9.7 ± 4.5		
TMT-A	154.4 ± 65.9		
Drainage volume of CSF	38.3 ± 3.5		
CSF opening pressure (cm H_2_O)	8.9 ± 3.0		
Evans’ ratio	0.32 ± 0.01		

Values denote number (%) or mean ± standard deviation.

INPH = idiopathic normal-pressure hydrocephalus; CSFTT = cerebrospinal fluid tap test; K-MMSE = Korean version of Mini-Mental State Examination; CDR = Clinical Dementia Rating Scale; INPHGS = Idiopathic Normal-Pressure Hydrocephalus Grading Scale; GS-Gait = INPHGS for gait; GS-Cogn = INPHGS for cognition; GS-Urin = INPHGS for urinary function; TUG = Timed Up-and-Go test; GSS = Gait Status Scale; FAB = Frontal Assessment Battery; TMT-A = Trail Making Test Part A.

### Shape Differences of the Lateral Ventricles between INPH Patients and Healthy Controls

Table 2 shows lateral ventricular volumes. Patients with INPH showed volume enlargements in the bilateral lateral ventricles relative to healthy individuals after covarying for age and intracranial volume.

**Table 2:** Volumetric measurements of brain MR Imaging.

Structure	INPH (n = 33)	Control (n = 23)	*P* value
Intracranial volume (cm^3^)	1151.5 ± 135.1	1078.5 ± 95.2	0.008
Right lateral ventricle (mm^3^)	56257.0 ± 20333.8	14857.7 ± 6980.8	<0.001
Left lateral ventricle (mm^3^)	62871.9 ± 23843.3	17502.4 ± 7448.8	<0.001
Total lateral ventricle (mm^3^)	119128.9 ± 43087.7	32360.1 ± 14232.6	<0.001

Values denote mean ± standard deviation.

INPH = idiopathic normal-pressure hydrocephalus.

**Figure 1:**
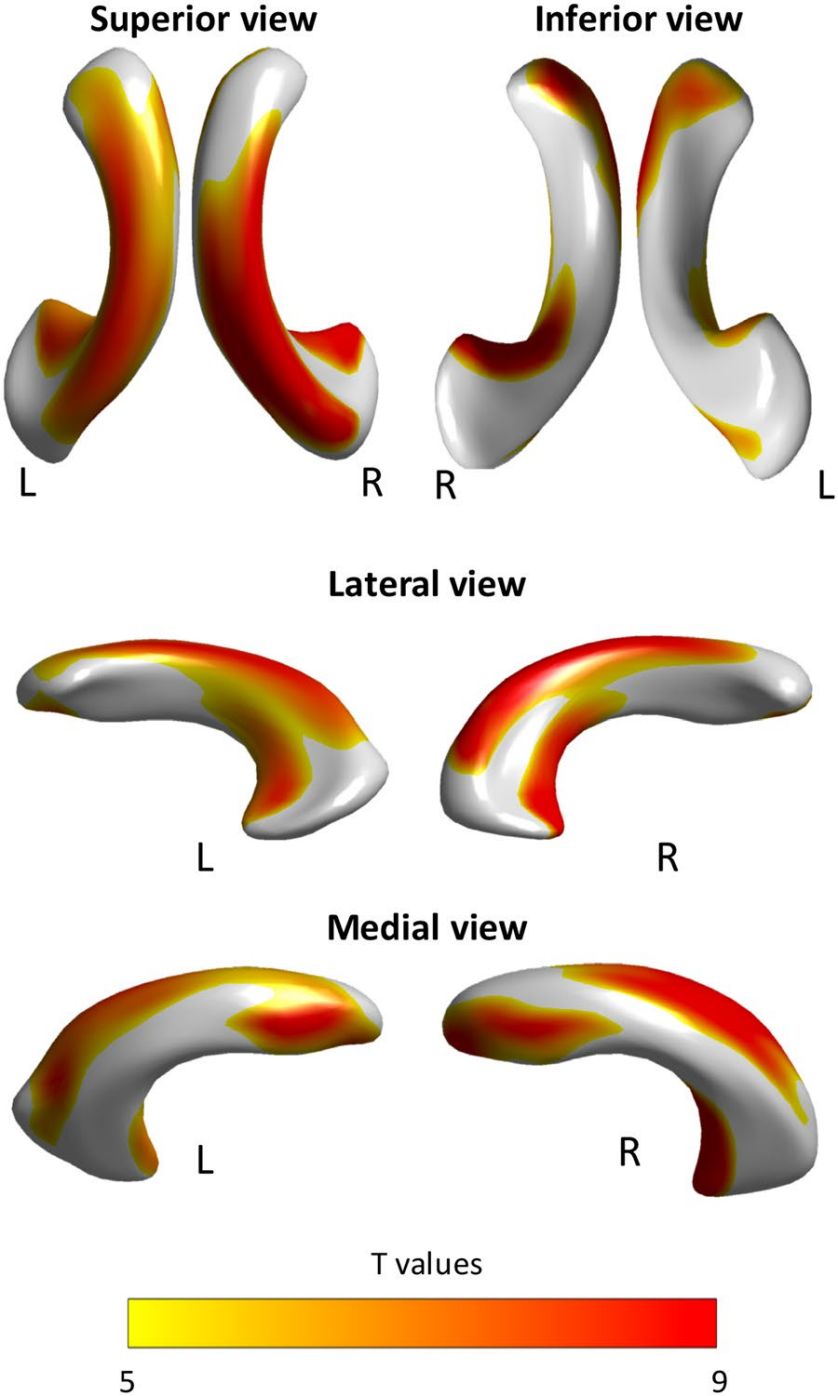
Statistical maps illustrating the location of shape differences in the lateral ventricles between the INPH and control groups. Clusters illustrated in the figure denote the significant surface expansion in INPH patients relative to controls following a Bonferroni correction for multiple comparisons.

Surface maps demonstrating significant surface expansion of the lateral ventricles in the INPH group relative to the control group are presented in Fig. 1. All results were adjusted for age and intracranial volume. A significant surface expansion in the INPH group was observed mainly in the clusters located in the superior portion of the bilateral lateral ventricles, which are adjacent to the high convexity of the frontal and parietal regions. The inferior portion of the bilateral lateral ventricles appeared much less affected. INPH patients showed a significant expansion of the medial aspects of the frontal horns of the bilateral lateral ventricles to the surrounding brain regions, including the medial frontal lobe. The temporal horns of the lateral ventricles, which are located near the medial temporal lobe structures, were also expanded in the INPH group relative to the control group.

### Correlations of Lateral Ventricular Expansion and Cortical Thinning

**Figure 2:**
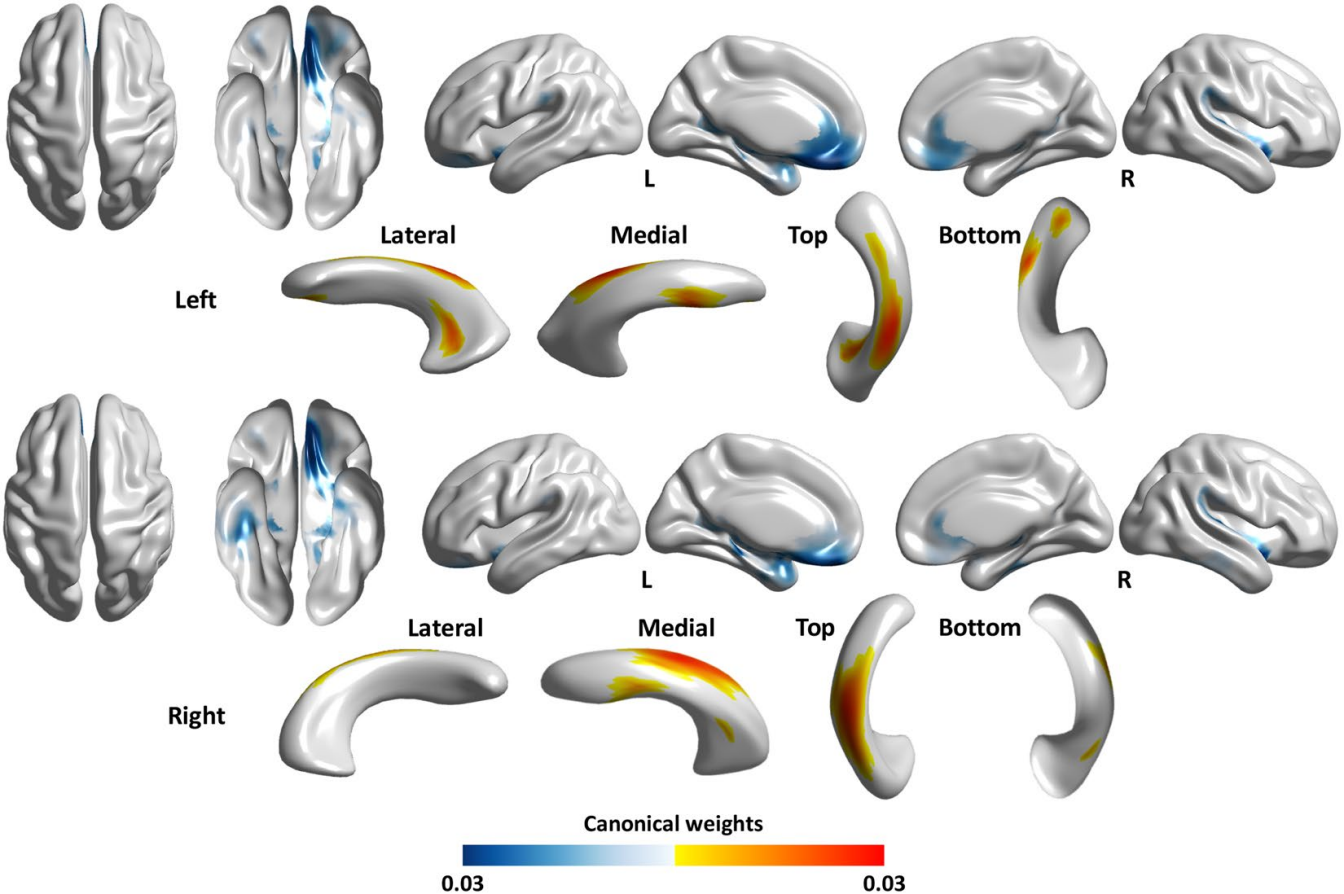
Statistical maps showing the correlation between lateral ventricular expansion and cortical thinning in INPH patients. A higher canonical weight number indicates higher correlation. Warm color (red-yellow) indicates larger lateral ventricular expansion while cold color (blue-light blue) indicates cortical thinning in INPH compared to the controls.

Our results in Fig. 2 indicated that the ventricular surface expansion, particularly noticeable in the superior portion, was associated with cortical thinning in the bilateral orbitofrontal cortex and bilateral rostral anterior cingulate cortex. The ventricular surface expansion was also associated with cortical thinning in the left parahippocampal cortex, left temporal pole, right insula, right inferior temporal cortex, and right fusiform gyrus. Overall cortical thickness did not differ significantly between the two groups (mean overall cortical thickness in the INPH group = 2.83 ± 0.19 mm, in the healthy control group = 2.88 ± 0.16 mm, p = 0.077).

## Discussion

Compared with age- and gender-matched healthy controls, INPH patients showed a significant surface expansion mainly in areas located in the medial aspects of the frontal horns and the superior portion of the bilateral lateral ventricles, which are surrounded by the high convexity of the frontal and parietal regions and the medial frontal lobe. Additionally, the temporal horns of the bilateral lateral ventricles, which are surrounded by the medial temporal lobe structures, were expanded in the INPH group relative to the control group. These results provide some evidence for a characteristic pattern of lateral ventricular enlargement in INPH patients.

As an explanation for the lateral ventricular expansion in the medial aspects of the frontal horns and the superior portion for INPH patients, we may speculate as follows. Morphologically distinctive features of the CSF spaces have been reported in INPH. While patients with INPH show marked ventricular dilatation, narrowing of the CSF space at the high convexity and high midline areas has been suggested to be one of the characteristic imaging findings in INPH^45,46^. It has been suggested that the characteristic narrowing of the CSF space at the high convexity/midline may be caused by defective CSF absorption as a result of suprasylvian subarachnoid block^47^. The blockage of the suprasylvian CSF subarachnoid space may produce a compartmentalized gradient of pressure in this space^47^. A high pressure in the ventricles and a low pressure in the suprasylvian subarachnoid space are regarded as prerequisites for lateral ventricular enlargement and a constricted suprasylvian subarachnoid space^46^. However, the finding that the size of the sylvian fissure diminished together with ventricular size after shunt surgery suggested a balanced pressure between these two compartments^46^. Regionally-specific patterns of the affected lateral ventricles might be subordinate to the pressure gradients across the ventricular system and the suprasylvian subarachnoid spaces. Our finding, that the inferior portion of the bilateral lateral ventricles appeared much less affected in INPH, is also consistent with these previous results.

As potential explanations for the lateral ventricular expansion in the temporal horns for INPH patients, we may offer the following suggestion. Dilatation of the temporal horns of the lateral ventricles is well known to be an established finding in hydrocephalus^48^. Wide temporal horns are common in patients with INPH and were regarded as significant predictors of a positive shunt outcome^48^. Dilatation of the temporal horn is also known as one of the earliest signs of hydrocephalus^49^. Furthermore, the medial temporal lobe structures have been identified as the earliest and the most severely affected structures in the neuropathology of the AD^50^. The lateral ventricle, especially the temporal horn, is also a structure of interest in the study of AD given its spatial adjacency to the medial temporal lobe structures^50^. Multiple publications have reported enlargement of the temporal horns of the lateral ventricles in AD^51,52^. Some of these authors have further suggested that it might be an accurate marker of the disease^51,52^. It appears that there should be dilatation of the temporal horns in both INPH and AD^49^. AD is frequently concomitant with INPH, with one study finding that 89% of INPH patients exhibited AD pathology^53^. Taken together, comorbid neurodegenerative pathology, such as AD, may further exacerbate or contribute to dilatation of the temporal horns in INPH. Further studies on INPH patients using pathophysiological biomarkers associated with AD would be needed to establish this hypothesis.

In this study, ventricular surface expansion, particularly noticeable in the superior portion, significantly correlated with cortical thinning in the bilateral orbitofrontal cortex and bilateral rostral anterior cingulate cortex. Concerning cerebral blood flow (CBF) in INPH, previous studies have frequently reported that frontal-dominant perfusion decreases using single photon emission computed tomography or positron emission tomography^54,55^. Ventriculomegaly seems to compromise the vascular supply to specific brain regions^56^. It has been suggested that distortion of the brain parenchyma certainly does occur in INPH and distortion rather than adjacent compression may be more directly associated with a reduction in CBF^55^. Interestingly, paradoxically increased CBFs in the high convexity of the frontal and parietal regions were observed in the INPH group in comparison to the control group^57^. Furthermore, cerebral hypoperfusion has been considered as a predictor variable for cortical thinning^58^. In this study, SCCA revealed unique relationships between lateral ventricular enlargement and cortical thinning in INPH patients. However, the question remains: what mechanisms underlie the connection among ventricular enlargement, brain distortion, and CBF reduction? The links among these would serve as an intriguing area for future research.

In our study, ventricular surface expansion also significantly correlated with cortical thinning in the left parahippocampal cortex, left temporal pole, right insula, right inferior temporal cortex, and right fusiform gyrus. How might the association between these areas be explained? We may offer a possible scenario. It has been suggested that CSF stasis, in humans as well as in animals, promotes amyloid deposits mainly in older subjects^59^. AD and INPH are reported to have a common physiological basis in CSF circulatory dysfunction and failure^60^. Failure of the CSF to clear potentially toxic metabolites would lead to accumulation of amyloid peptide in the brains of patients with AD or INPH^60^. The parahippocampal cortex, temporal pole, insula, inferior temporal cortex, and fusiform gyrus are typically thought to be affected earliest in the course of AD on the basis of the burden of pathologic accumulation^61–63^. The volume of CSF is known to be a substantial factor in the CSF turnover rate^64^. Large ventricles inherently impair the CSF’s ability to efficiently renew itself because turnover rate is inversely related to CSF volume^64^. Accordingly, in INPH, the CSF turnover rate can fall by 3-4-fold^64^. And as the ventricles enlarge in INPH, the purity of CSF is gradually lost due to less rapid turnover^64^. We cautiously suggested that the CSF stasis caused by ventricular expansion in INPH patients may promote AD-like structural brain changes.

INPH subjects were selected in consecutive order from our prospectively enrolled INPH registry. We tried to reduce the potential bias from clinical evaluation before and after CSFTTs by using various and objective grading scales, instead of subjective reports by patients or caregivers. In addition to a small sample size, one limitation of this study was that we did not include INPH patients who had a negative response to the CSFTT. However, we were motivated to enhance diagnostic certainty of INPH by restricting our study to CSFTT responders. Additionally, INPH patients with a negative response to the CSFTT were more likely to have other cerebral comorbidities^65^. A second limitation was that we did not measure CBF and AD-specific biomarkers in our study. As a result, both CBF reduction and AD pathology could not be confirmed in our INPH patients. Nevertheless, in our study, we also believe that there might be merit in utilizing three-dimensional, surface-based, morphometric analysis along with SCCA in a relatively large sample of INPH patients. To our knowledge, there is no study about the three-dimensional, surface-based, morphometric analysis of the lateral ventricles in INPH.

In conclusion, morphometrically distinctive features in lateral ventricles were found in INPH patients. The INPH patients in our study showed a significant surface expansion primarily located in areas adjacent to the high convexity and high midline of the frontal and parietal regions. The areas located near the medial temporal lobe structures were also expanded in the INPH group relative to the control group. Further, this study additionally showed that ventricular surface expansion correlated with cortical thinning in the orbitofrontal cortex, rostral anterior cingulate cortex, parahippocampal cortex, temporal pole, insula, inferior temporal cortex, and fusiform gyrus. Our findings encourage future studies to elucidate the underlying mechanism of lateral ventricular morphometric abnormalities in INPH patients.

## Data Availability

The datasets generated and analysed during the current study are available from the corresponding author upon reasonable request.
